# Reverse Magnetization Behavior Investigation of Mn-Al-C-(α-Fe) Nanocomposite Alloys with Different α-Fe Content Using First-Order Reversal Curves Analysis

**DOI:** 10.3390/nano12193303

**Published:** 2022-09-22

**Authors:** Seyed Nourallah Attyabi, Seyyed Ali Seyyed Ebrahimi, Zahra Lalegani, Bejan Hamawandi

**Affiliations:** 1 Advanced Magnetic Materials Research Center, School of Metallurgy and Materials, College of Engineering, University of Tehran, Tehran 111554563, Iran; 2 Department of Applied Physics, KTH Royal Institute of Technology, SE-106 91 Stockholm, Sweden

**Keywords:** reverse magnetization, recoil curve, FORC analysis, Henkel plot, MnAlC

## Abstract

The reverse magnetization behavior for bulk composite alloys containing Mn-Al-C and α-Fe nanoparticles (NPs) has been investigated by hysteresis loops, recoil, and first-order reversal curves (FORC) analysis. The effect of adding different percentages of α-Fe (5, 10, 15, and 20 wt. %) on the magnetic properties and demagnetization behavior of Mn-Al-C nanostructured bulk magnets was investigated. The fabricated nanocomposites were characterized by XRD and VSM for structural analysis and magnetic behavior investigations, respectively. The demagnetization curve of the sample Mn-Al-C-5wt. % α-Fe showed a single hard magnetic behavior and showed the highest increase in remanence magnetization compared to the sample without α-Fe, and therefore this combination was selected as the optimal composition for FORC analysis. Magnetic properties for Mn-Al-C-5 wt. % α-Fe nanocomposite were obtained as *M_s_* = 75 emu/g, *M_r_* = 46 emu/g, *H_c_* = 3.3 kOe, and *(BH)_max_* = 1.6 MGOe, indicating a much higher *(BH)_max_* than the sample with no α-Fe. FORC analysis was performed to identify exchange coupling for the Mn-Al-C-0.05α-Fe nanocomposite sample. The results of this analysis showed the presence of two soft and hard ferromagnetic components. Further, it showed that the reverse magnetization process in the composite sample containing 5 wt. % α-Fe is the domain rotation model.

## 1. Introduction

Magnetic nanocomposites are one of the most promising ways to achieve a new generation of permanent magnets. In the last few decades, magnetic nanocomposites have attracted a lot of attention due to their high coercivity and improved remanence on a single domain scale. So far, Nd_2_Fe_14_B/α-Fe, SmCo/α-Fe, etc., alloys have been extensively studied due to their high coercivity [1,2,3,4,5,6].

In the early 1990s, Kneller and Hawig [7], and subsequently Skomski and Coey [8], re-examined the experimental work of Coehoorn et al. [9,10]. They showed that at the nanoscale, the soft ferromagnetic phase (high magnetism) with the hard ferromagnet (high coercivity) can produce magnetic composite materials with a further improvement of (*BH*)*_max_*. In this case, interphase magnetic exchange coupling is created in a composite system that takes advantage of the best characteristics of the constructive phases. Hard magnetic phases provide high anisotropy with high coercivity field, whereas soft magnetic phases provide high saturation.

The use of low-cost and plentiful elements is effective in reducing the cost of magnet production, which is very important for the development of permanent magnets in some applications. However, their magnetic properties are still much lower than those of Nd_2_Fe_14_B magnets. L1_0_MnAlC-(α-Fe) nanocomposite is one of the promising systems without rare earth elements that may be a good candidate for spring exchange magnet [11,12]. Mn-Al-C alloys have large exchange lengths and wide domain walls [13,14].

Therefore, it is important to find a way to increase the coercivity and remanence and to understand the mechanisms of reverse magnetization in the Mn-Al-C-(α-Fe) nanocomposite. There are numerous reports of increased coercivity by milling MnAl and MnAlC alloys fabricated by different methods [15,16,17,18,19,20].

Mechanical milling increases coercivity by decreasing grain size and increasing defects, but often the saturation magnetism and remanence decrease due to phase decomposition, formation of defects, and reduction in chemical order. However, using annealing, its magnetism can be restored due to an increased chemical order and reduced defects [21,22,23,24].

Studies that report on the reverse magnetization behavior of Mn-Al-C-(α-Fe) nanocomposites prepared by mechanical milling are very limited [14,22,25]. According to reports, the *(BH)_max_* of Mn-Al-C systems is typically below 1MGOe.

In this paper, we report the effect of Fe addition on the magnetic properties and reverse magnetization behavior of Mn-Al-C-(α-Fe) alloy composites prepared by mechanical milling method using FORC analysis. To the best of our knowledge, no studies on FORC analysis of Mn-Al alloys have been published. We were able to obtain a high *(BH)_max_* (1.6 MGOe) and thus take an important step in improving the magnetic properties of hard magnets without rare earth elements.

## 2. Experimental

### 2.1. Materials and Instrumentation

In this study, the impact of adding different amounts of α-Fe on the magnetic properties and demagnetization (reverse magnetization) behavior of Mn-Al-C nanostructured bulk magnets was investigated. For this purpose, α-Fe powders (purchased from US Research Nanomaterials, Inc., Houston, TX, USA) with a particle size of 35–45 nm were used. Further, a cyclohexane medium was used for experiments to prevent oxidation.

The actual composition of the cast alloys was determined by inductivity coupled plasma-optical emission spectroscopy (ICP-OES, Varian 730-ES, Palo Alto, Santa Clara, CA, USA) and the amount of carbon was measured using the LECO CS-244 carbon determinator (ASTM E1019). A high-energy planetary ball mill (Fritsch Pulverisette, model 6) with hardened chrome steel balls was used to grind the cast ingots. The phase analysis of the final nanocomposite samples was investigated using an x-ray diffractometer (XRD, Rigaku-Dmax 2500, Rigaku Corporation, Tokyo, Japan) with Cu-Kα radiation (λ = 1.54 $\dot{A}$) and 2θ range from 10° to 100°. Reverse magnetization behavior and FORC analysis of composite alloys were investigated by a vibrating sample magnetometer (VSM, Meghnatis Daghigh Kavir Co.; Kashan Kavir; Iran) at room temperature with maximum applied field of 15 kOe.

### 2.2. Mn_52_Al_45.7_C_2.3_ Nano Powder Fabrication

The alloy with nominal composition Mn_52_Al_45.7_C_2.3_ (at. %) was prepared by a vacuum induction melting process and was cast into a copper mold. Before the melting, due to the high vapor pressure of Mn, an extra 3 wt. % Mn was added. The re-melting process was performed twice under a controlled atmosphere (Ar) to ensure the homogeneity of the cast alloy and also to adjust the composition of the main alloy. Then, the resultant ingot was homogenized in a vacuum furnace (P = 1 ∗ 10^−4^ Torr) at 1000 °C for 5 h. Afterward, it was cooled in air to obtain the ferromagnetic τ phase. Finally, Mn_52_Al_45.7_C_2.3_ ingot was milled by high-energy planetary milling in a hardened-steel chamber (volume 100 cc) and ball size 5 mm in cyclohexane medium as a surfactant with a ball-to-powder weight ratio of 20:1 for 5 h. The Mn_52_Al_45.7_C_2.3_ nano powder with an average particle size of 100 nm was obtained (see Supplementary Materials, Figure S1).

### 2.3. Processing of Nanostructured Bulk Magnets

Mn_52_Al_45.7_C_2.3_ nano powders were mixed with different weight ratios of α-Fe powder (0–20 wt. %). The mixture was mechanically milled in a cyclohexane medium for 1 h to obtain a completely homogeneous composite powder. The composite powder was placed in a tungsten carbide mold with a diameter of 5 mm and pressed under a constant uniaxial pressure of 5.1 GPa. Afterward, the obtained bulk sample was annealed in a vacuum tube furnace at 400 °C for 30 min in order to achieve optimal magnetic properties. The samples with different weight percentages of α-Fe NPs are listed in Table 1.

## 3. Results and Discussions

### 3.1. Structural Analysis

**Figure 1:**
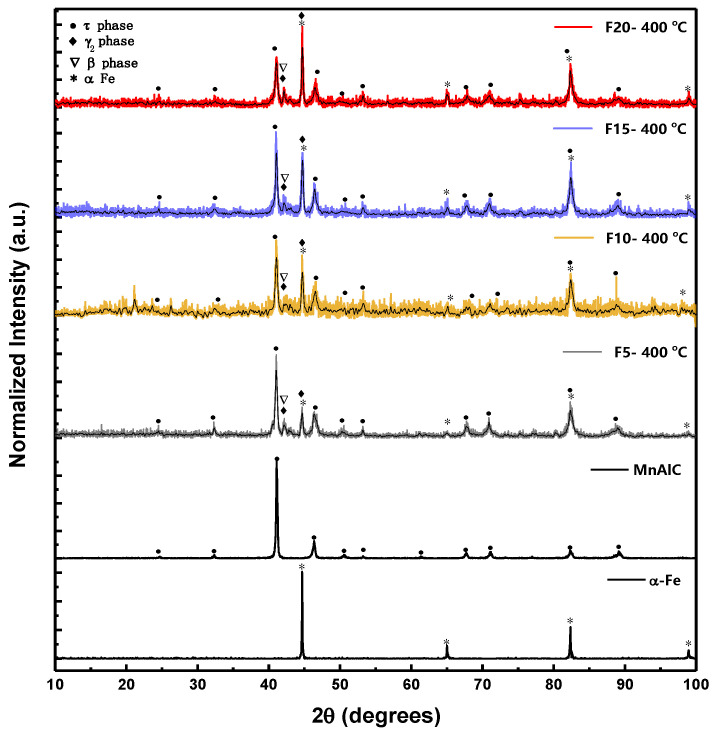
XRD patterns of Fe NPs, Mn-Al-C alloy, and magnetic composite samples.

The XRD patterns of Mn-Al-C alloy and Fe NPs as well as Mn-Al-C-(*X* wt. % α-Fe) magnetic composite NPs with *X* = 0 to *X* = 20 are shown in Figure 1. The Mn-Al-C alloy is homogenized at 1000 °C for 5 h and then cooled in the air to room temperature. The pure τ phase was identified according to the diffraction pattern of this alloy. The XRD pattern of Mn-Al-C and α-Fe NPs is in good agreement with the JCPDS card no. 00-037-1073 and 00-006-0696, respectively.

It can be seen that all XRD patterns of magnetic composite samples show a hard magnetic phase (L10 tetragonal P4/mmm space group (#123)) and a soft magnetic phase of α-Fe (space group Im-3m (229)). The τ-MnAlC phases are metastable and after two millings, some low intensity peaks with non-magnetic phases of β-Mn_3_Al_2_ (cubic space group p4132 (213), JCPDS card no. 00-048-1568) and γ2-Al_8_Mn_5_ (rhombohedral space group R3m (160), JCPDS card no. 00-032-0021) were observed. Further, in the XRD pattern of composite NPs, the peak 2θ = 44.67° corresponding to the plane (1 1 0) of the BCC phase has increased with increasing α-Fe concentration.

Moreso, the addition of α-Fe NPs had no effect on the phase structure in these alloys and no mechanical alloying occurred.

### 3.2. Magnetic Properties

#### 3.2.1. Hysteresis Loops Investigation

**Figure 2:**
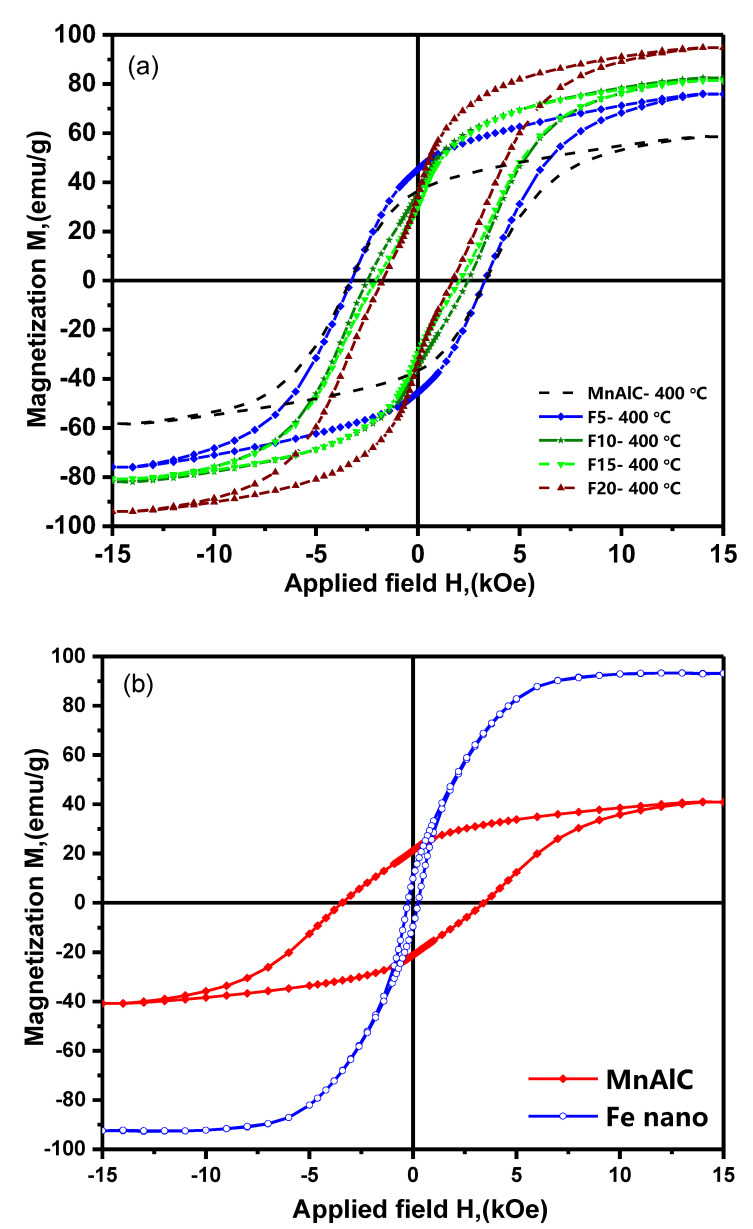
(**a**) Hysteresis loops of Mn-Al-C alloy, Mn-Al-C-α-Fe nanocomposite alloys with different amounts of α-Fe annealed at 400 °C for 30 min; (**b**) hysteresis loops of α-Fe and Mn-Al-C alloy without annealing.

Magnetic measurements were performed at room temperature by VSM with a magnetic field of 15 kOe. Figure 2 shows the hysteresis loops of ferromagnetic Mn-Al-C alloy, Mn-Al-C-α-Fe nanocomposite alloys with 5–20 wt.% α-Fe (annealed at 400 °C for 30 min), and α-Fe.

According to Figure 2, the *M(H)* demagnetization curve of the F5 sample in the second quarter, between *M_r_* and *M* = 0, is convex similar to that of the Mn-Al-C single-phase permanent magnet. This indicates that the demagnetization curve of the Mn-Al-C-0.05α-Fe alloy has a single hard magnetic behavior. It means the hard magnetic phase is uniformly under the reversed external magnetic field and indicates the hard and soft magnetic phases have good exchange coupling.

However, for samples F10, F15, and F20 the demagnetization curve is concave. In both hard and soft magnetic nanocomposites, a constricted loop is obtained if there is no exchange coupling. In this case, a kink is created in the curve [7].

According to Figure 2, it can be inferred that the optimal magnetic properties belong to the sample containing 5 wt.% α-Fe, because in this sample there is the highest increase in remanence magnetization compared to the sample without α-Fe. In addition, the maximum energy product is (*BH*)*_max_* = 1.6 MGOe, which is an increase of 60% compared to the Mn-Al-C sample. Increasing the saturation magnetization (*M_s_*), the remanence magnetization (*M_r_*), and the squareness (*M_r_*/*M_s_*), and thus the increase in (*BH*)*_max_* for 5 wt% α-Fe sample compared to the sample without α-Fe can be due to the uniform distribution of soft and hard phases, and the uniform NPs size of Mn-Al-C and α-Fe phases. It can also be a sign of a strong exchange coupling effect. The obtained magnetic properties for Mn-Al-C-(0.05α-Fe) alloy are more improved properties compared to the properties reported in the literature. A comprehensive comparison of the properties reported on the Mn-Al alloy with the present work is also given in Table 2. Since there is an exchange coupling between the hard phase and the soft phase, crystallographic coherency between the two phases is required. This means that the easy magnetization axis directions in the hard and soft phases are parallel. Therefore, the remanence magnetization for the soft phase (*M_rs_*) and for the hard phase (*M_rh_*) are aligned [7,26,27].

One of the reasons for the increase in squareness *M_r_*/*M_s_* is the existence of exchange couplings between particles. Based on the model proposed by Kneller [7], exchange coupling of soft phase (with cubic structure) and hard phase (with tetragonal/hexagonal structure) in polycrystalline samples containing independent magnetic grains with random magnetic orientation occurs when the easy magnetization axis directions in the soft and hard phases are perfectly aligned, and in this case the relative residual is reported to be *m_r_* = *M_r_*/*M_s_* ≥ 0.68. According to our results, its value for F5 sample is 0.61, which indicates that the easy magnetization axis direction in many soft phases is aligned with the easy direction in hard phases. For other samples containing more than 5 wt. % α-Fe, this value is much less than 0.5. The decrease in *M_r_* and *H_c_* is due to the increase in the soft phase; and due to the occurrence of soft/hard biphases, it is clearly visible in the hysteresis loops of Figure 2 in the form of kinks. This low value of squareness can be attributed to the high content of soft phase NPs in the composite and possibly the agglomeration of soft phase particles, which prevents the exchange coupling with the hard phase and increases the magnetostatic interaction, thus weakening the exchange interactions. According to Coey’s theory of magnetic exchange couplings [8], the size of soft phase should not be more than twice the wall thickness of hard phase, which is usually about a few tens of nanometers. According to the calculations of Luo et al. [13], the Mn-Al domain wall thickness was announced as 10.9 nm. Coey and Skomski [8] stated that fine particles of soft phase retain their coercivity, and as the particle size increases, the coercivity decreases by 1/*D^2^* due to the presence of heterogeneous magnetostatic fields.

In 2013, Skomski et al. [99] performed a simulation on soft nanostructures and concluded that the presence of a soft phase in the hard phase matrix is relatively better than the hard geometry in a soft one, and embedded soft phase particles are better than sandwiched soft layers, and the nucleation field is a function of the soft phase size. The embedded soft spherical particles have the highest coercivity. For very small soft particles, the nucleation field is close to the anisotropic field of the hard phase.

According to Figure 2, by adding α-Fe, the coercivity and squareness remained constant whereas the saturation and remanence magnetization increased, and with increasing the amount of α-Fe soft phase, the values of coercivity, squareness, and remanence magnetization decreased. The saturation magnetization of the F20 sample is almost identical to the saturation magnetization of the α-Fe soft phase. Therefore, the main challenge is the uniform distribution of the soft phase in the hard phase in order to maintain *H_c_* and achieve a high (*BH*)*_max_*.

#### 3.2.2. Switching Field Distribution Curves Investigation

**Figure 3:**
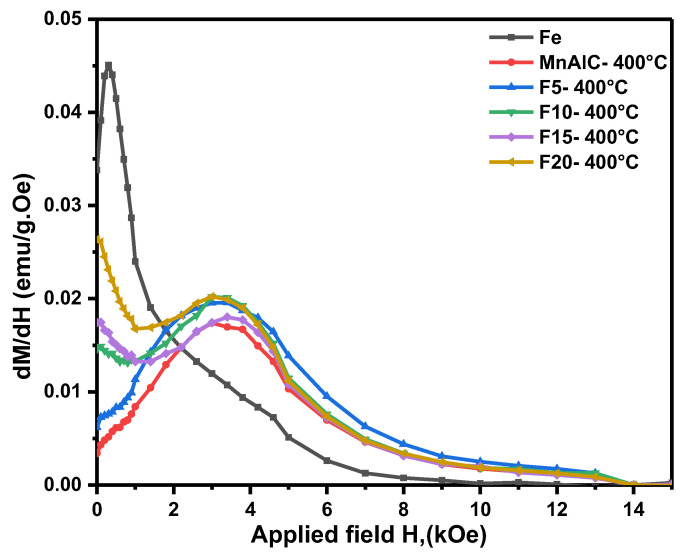
Switching field distribution curves of samples.

Magnetic exchange coupling can be proved by a kink-free smooth hysteresis loop. The smoothness of the hysteresis loop can be evaluated by the hysteresis derivative (*dM*/*dH*) vs. *H* curve known as the switching field distribution curve (Figure 3). The switching field distribution is known as the phase-exchange property and indicates the particles coercivity distribution in the samples [100,101,102].

According to Figure 3, two peaks are observed for samples F10, F15, and F20 that confirm the biphase magnetic behavior. A peak appears near the zero field (*H* = 0), which has grown as the amount of soft magnetic phase in the nanocomposites increases. This means that the α-Fe phase is not associated with a good exchange coupling. According to Figure 3, the second peak is a wide peak that indicates the coercivity of hard magnetic NPs. The presence of a single peak for F5 composite NPs is similar to the behavior of hard magnetic single phase Mn-Al-C, indicating reverse magnetization in one stage and hard/soft phase exchange coupling. The switching field distribution curve peak of the F5 sample is located in the 3.3 kOe field, whereas this peak for the Mn-Al-C sample is in the 2.9 kOe field.

As can be seen in Figure 3, the coercivity distribution of sample F5 is wider than the Mn-Al-C (i.e., wider peak), which is a sign of weakness in exchange coupling. The peak of sample F5 extends from both sides of the lower and higher coercivity fields and is elongated. One of the main reasons for the expansion to the lower fields is the presence of a soft phase, and given that *K_s_* < *K_h_*, in the lower applied field it seeks to rotate. Although the hard phase acts as a barrier and prevents the rotation of the soft-phase magnetic domains in the low field, due to the size of the α-Fe soft phase NPs being larger than the critical size, some domains in the lower field have switched to the applied field.

Additionally, the presence of soft phases in the hard phase matrix reduces the antiferromagnetic coupling between the Mn–Mn pair at the interphase boundaries and leads to an increase in the coercivity of the hard NPs; hence, the switching field distribution has elongated to the higher fields. In addition, in samples F10, F15, and F20, the switching field distribution is extended to higher coercivity fields, but due to the formation of biphase magnetic particles, the coercivity in the samples has decreased.

#### 3.2.3. Henkel Plots Investigation

To study and determine the mechanism of interaction between magnetic composite NPs and to confirm the interparticle exchange coupling, the samples were investigated by the Henkel plot (*δM*—*H* curve). The *δM*—*H* curve provides qualitative information about the interactions of magnetic NPs with the following equation [53,103,104,105]:

*δM = M_d_ (H) − [1 − 2M_r_ (H)]* (1)

where *M_d_(H)* is the normalized demagnetization remanence, *M_r_(H)* is the normalized isothermal remanent magnetization, and *H* is the applied magnetic field.

*M_r_(H)* was measured by gradually increasing and removing the magnetic field (*H* > 0), and this was repeated until saturation magnetization (*H* = *H_sat_*). *M_d_(H)* was also measured after saturation of the sample and removal of *H_sat_*, by applying and removing a negative magnetic field (*H* < 0) and finally normalized by *M_r_(H_sat_)*.

According to Wohlfarth’s analysis, *δM* > 0 indicates a ferromagnetic exchange coupling between the hard and soft phases in the system, whereas *δM* < 0 is due to the bipolar interactions (magnetostatic interactions).

**Figure 4:**
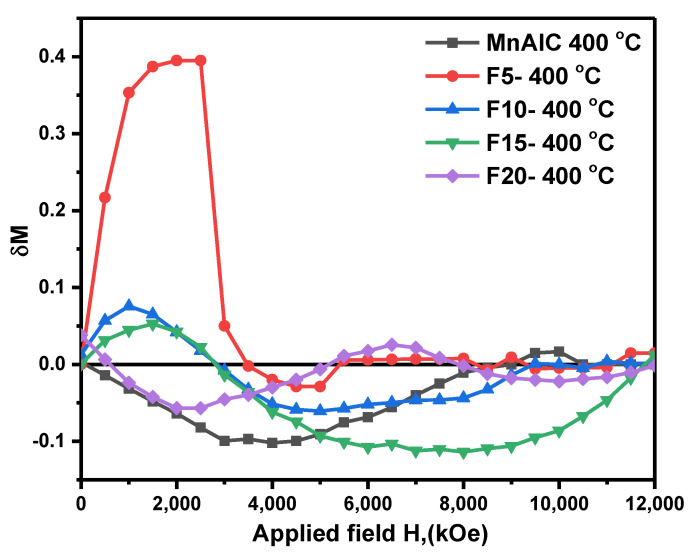
Henkel plots of the samples.

Figure 4 shows the Henkel plots of the samples. As shown in Figure 4, for the Mn-Al-C sample, *δM* is negative, indicating the predomination of interparticle magnetostatic interactions and no magnetic exchange coupling in the sample. In the F5 curve, there is a peak in the positive region of *δM*, which indicates the dominance of exchange coupling interactions. A slight negative peak can also be seen in the 3.5 kOe–5.5 kOe field, indicating a weak magnetostatic interaction in the sample, which has caused the switching field distribution to expand. With an increasing soft phase, the negative *δM* peak becomes more pronounced and the positive peak height decreases, which indicates a decrease in magnetic exchange coupling and the predominance of strong interparticle magnetostatic interactions, leading to the destruction of magnetic properties. The maximum positive peak height of *δM* is for the F5 sample, which confirms that the strongest interparticle exchange coupling is obtained for this sample, leading to optimal magnetic properties.

#### 3.2.4. Recoil Curves Investigation

**Figure 5:**
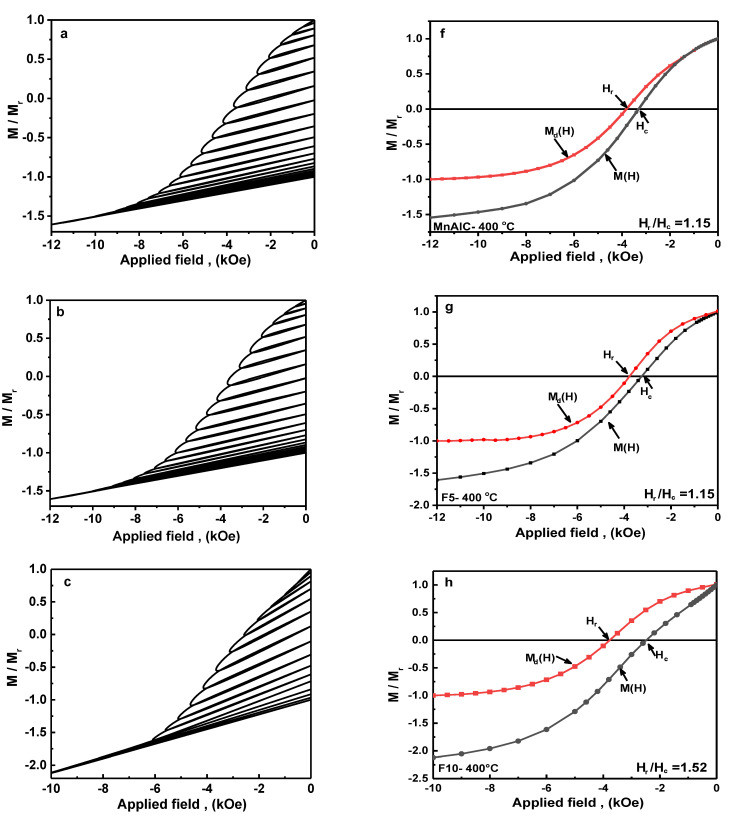

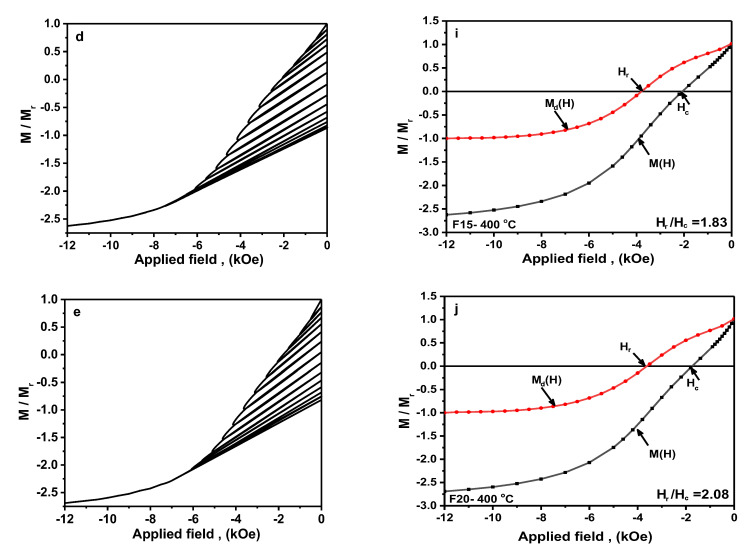
(**a**–**e**) Recoil demagnetization curves, (**f**–**j**) magnetization curves *M(H)* and dc demagnetization remanence *M_d_(H)* vs applied reversal field.

The recoil demagnetization curves are plotted in Figure 5 to better understand the reverse magnetization behavior of the samples. For this purpose, a 12 kOe magnetic field was applied and then reduced to zero to obtain *M_r_*. The reversed field (*H_d_*) was then applied and then reduced to zero. Magnetization under zero field after applying *H_d_* (*M_d(H)_*) was measured and this method was repeated until *H_d_* = 12 kOe. As shown in the Figure 5a,b, the recoil curves of the Mn-Al-C and F5 samples are very similar.

The magnets with highly reversible magnetic behaviors are called exchange-spring magnets [7,106,107]. By increasing the soft phase in composite, the reversibility increases and a high remanent reversible score in the sub-coercive fields can be seen for the F20 sample, but because *M_r_*/*M_s_* < 0.5, they cannot be considered as exchange-spring magnets. With increasing in the amount of soft phase, the curves in Figure 5 change from convex to concave. It indicates that the size of NPs in the soft phase is close to the critical size (b_s_ ≈ b_cs_) in the F5 sample. Therefore, this sample has an optimal microstructure, and increasing the number of soft phases and their accumulation together causes to overaged microstructure. Therefore, the demagnetization curve is concave. The curves of F10 and F15 samples are slightly convex, which indicates that there is some exchange coupling in these magnets as well. This is consistent with the Henkel plots results of these samples.

According to Kneller and Hawig’s micromagnetic models [7], single-phase magnets always have a closed recoil loop due to reversible behavior, which can simultaneously indicate reverse magnetization and stronger exchange coupling. Meanwhile, according to the micromagnetic finite element model calculated by Zheng [108], the reduction in the recoil loop area can occur by reducing the soft magnetic phase. Recoil loop area in nanocomposite magnets is generally interpreted as failure in interparticle exchange coupling and is attributed to the reverse magnetization behavior in grain boundaries or uncoupled soft magnetic regions, where magnetic moments are unstable due to high exchange energy, and a distribution is observed in the hard magnetic anisotropy constant *K* [109]. However, in the SmCo/α-Fe and τ-MnAlC systems, it has been reported that the presence of an open recoil loop may not be due to failure in interphase exchange coupling [30,110]. The presence of open loops is not only due to the addition of soft magnetic single-phase in nanocomposite magnets, but also by the grain boundaries, grain size reduction, and distribution in the hard magnetic phase anisotropy constant *K*.

The magnetic phase of Mn-Al-C is a single-phase alloy with no soft magnetic phase, and has an open recoil loop, which near *H_c_*, the loop area reaches a maximum. Hence, changes in the hard magnetic particle anisotropy and a decrease in particle size could be responsible for the open recoil loop. If some Mn atoms are coupled antiferromagnetically (due to the high concentration of Mn with the formation of the Mn-rich τ-phase and also the presence of defects in the lattice), it is possible that the recoil loop has an area.

To better understanding of the interparticle coupling, the diagrams of *M_d_(H)* and *M(H)* are plotted as a function of the applied reversal field for samples Mn-Al-C and F5 (Figure 5f–n). *H_r_* is the dc remanence coercivity of which is determined by *M_d(Hr)_* = 0 and *H_c_* is the intrinsic coercivity which is determined by *M_(Hc)_* = 0 condition [7,111,112]. The *H_r_*/*H_c_* ratio for Mn-Al-C and F5 samples is 1.15 which is close to the theoretical value predicted by Wohlfarth, i.e., *H_r_*/*H_c_* = 1.09, whereas this ratio is much higher for other alloys [7,111]. These results show that the F5 alloy, like the Mn-Al-C, has a good single hard magnetic behavior.

According to the Stoner–Wohlfarth model for ideal single-domain grains, *H_c_* is equal to anisotropy field *H_a_*. In real magnets, *H_c_* is much lower than *H_a_*, and this is due to the less energy to reverse magnetism in the defects and the distribution of constant anisotropy. *H_a_* or *K* is basically related to the magnetization reversal stability in the grains. Grains with higher H_a_ can resist a stronger magnetizing field. Therefore, reversing the magnetization in defective areas (grains with less *H_a_*) can help to reverse the magnetization of grains with higher *H_a_* under less field and cause a sharp decrease in *H_c_*. On the other hand, the reversal of magnetization in the areas with accumulated α-Fe particles (less *H_a_*), could help to reverse the magnetization of τ-Mn-Al-C particles with higher *H_a_* under the lower field and cause a sharp decrease in *H_c_*. Therefore, the *H_rc_*/*H_c_* ratio is closely related to the reversal of magnetization and the resistance of the domains to rotation or domain wall movement due to the applied magnetic field.

**Figure 6:**
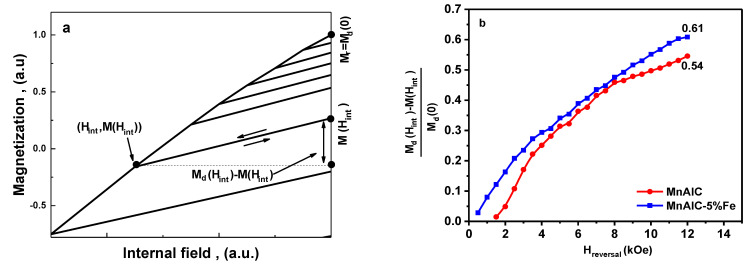
(**a**) Recoil curves showing critical parameters, and (**b**) comparison of reversible magnetization as a function of applied reversal field for Mn-Al-C and F5 samples.

**Figure 7:**
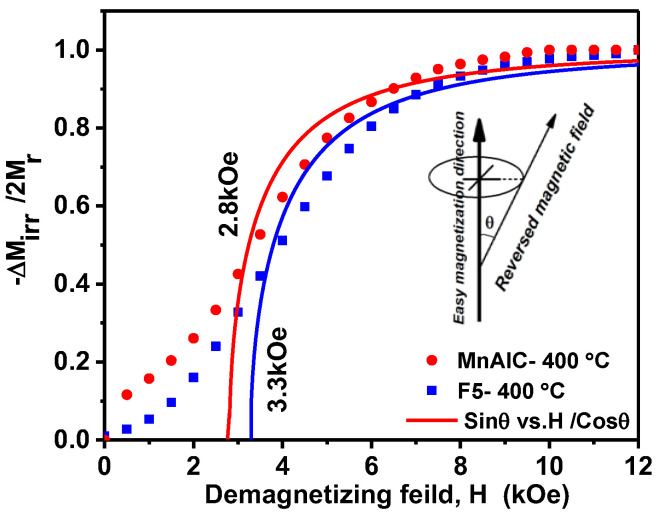
Irreversible magnetization vs applied reversal field for Mn-Al-C and F5 samples.

Hard single-phase ferromagnetics generally have a low reversible magnetism. To prove this property, recoil curves are used, and magnetic reversibility and irreversibility curves are plotted in Figure 6 and Figure 7. The magnetization is expected to be reversible in the presence of exchange coupling. The reversible process is associated with the rotation of the magnetism or the displacement of the domain wall in a single potential well [30]. Here the recoil curve used to determine critical parameters is shown in Figure 6a. Reversible magnetization changes (*M_d_*(*H_int_*) − *M*(*H_int_*)) were investigated by removing reverse magnetic field. Figure 6b shows the normalized recovered magnetization [*M_d_*(*H_int_*) − *M*(*H_int_*)]/*M_d(0)_* as a function of the applied reverse field (*H_reversal_*) for samples F0 and F5. The difference in magnetization in Figure 6 can be due to the interparticle exchange spring coupling behavior. According to Figure 6, the value obtained for sample F5 is 0.61, which is higher than 0.54 for sample Mn-Al-C. This indicates that there is a stronger spring exchange behavior in the F5 sample in comparison with the iron-free sample. Therefore, by adding 5% of the soft phase, the magnetic recovery is increased.

Irreversible changes are related to energy loss through the movement of the domain wall or the switching of magnetization in a single domain particle [113,114,115]. The irreversible part is described by the dc field demagnetization remanence (*M_d(H)_*), which remanence after saturation is in one direction and the dc field is in the opposite direction. The irreversible changes in the reverse magnetization process for the hard magnetic single-phase alloy Mn-Al-C and the hard/soft magnetic NPs Mn-AL-C/Fe as *D_(H)_* = [*M_r_* − *M_d(H)_*]/*2M_r_* ≈ − *ΔM_irr(H)_*/2*M_r_* were plotted versus *H* (Figure 7). It should be noted that the D(H) vs. H curve is measured in the optimum state of the alloy (isotropic grains) according to the one-dimensional purely inhomogeneous magnetization rotation model [7].

The total irreversible change in magnetization is as follows:

(2) $$\Delta M_{\mathrm{irr}}=2M_{r}\int_{0}^{\vartheta 1}cos\theta .d\theta =2M_{r}\sin\theta$$

*θ* is the angle between the field $\vec{H}$ and the easy axis of particles magnetization rotation, and $0\leq \theta \leq 90^{o}$ (with assumption of equal probabilities of all angles in the easy axis plane between the field $\vec{H}$ and the easy axis of magnetization rotation).

The negative applied external field required to destabilize the initial magnetization and initiate the reversal of the magnetization is defined as nucleation field *H_n_* [7]. When the reverse field *H* increases from *H* = 0 in the opposite direction, reverse magnetization does not occur irreversibly until *H* < *H_n_*. When *H* increases to *H_1_* = *H_n_*/*cos θ*, all particles reverse their magnetization by their easy magnetization axis at an angle θ relative to the applied field *H*. Equation (2) was written as a math function in the Origin software and fitting was done using the nonlinear curve fit option and the nucleation field was obtained. Curves fitting indicated that nucleation (switching) fields for Mn-Al-C hard single-phase magnetic alloy and F5 sample are 2.8 kOe and 3.3 kOe, respectively. Therefore, *H_n_* for sample F5 is higher than Mn-Al-C. The antiferromagnetic coupling of Mn–Mn may occur less at the phase boundaries and therefore the nucleation field may increase. The nucleation field close to the coercivity field (*H_n_*/*H_c_* ≈ 1) for sample F5 indicates the presence of effective exchange coupling in this sample, whereas *H_n_* is smaller than *H_c_* for Mn-Al-C, which indicates the presence of magnetostatic interactions.

#### 3.2.5. First Order Reversal Curves (FORC) Analysis

**Figure 8:**
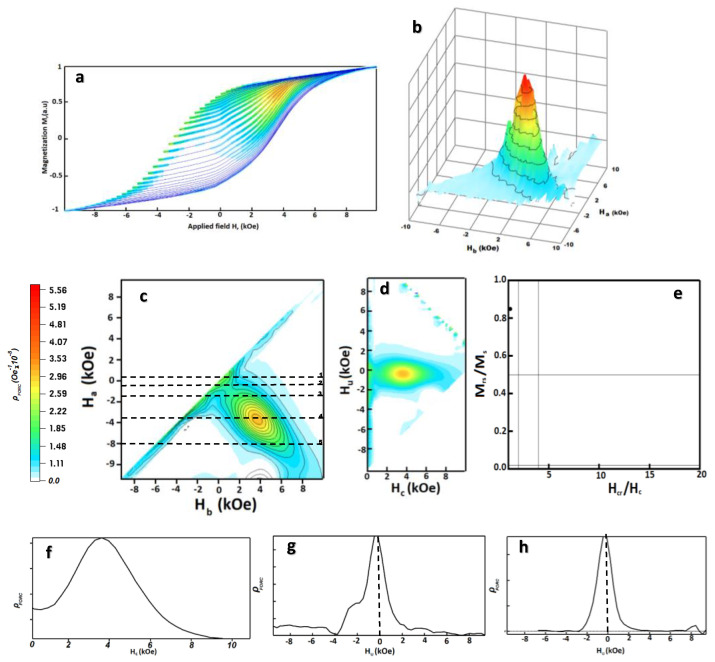
The calculated FORC analysis for sample F5 (milled and annealed at 400 °C); (**a**) a set of FORC measurements; (**b**–**d**) 3D and 2D diagrams calculated according to part a; (**e**) Day plot diagram; (**f**) cross section of the fork diagram with the maximum coercivity distribution (along the axis H_c_ = 0); (**g**,**h**) cross section of maximum interaction distribution along the axis H_u_ = 0 and H_u_ = 3.5 kOe.

In order to investigate the coercivity mechanism (demagnetization behavior) of F5 sample and identify the existing interaction field, FORC measurements were performed on this nanocomposite. Using FORC analysis, quantitative information can be obtained about the distribution of the coercivity field (*H_c_*) of each magnetic component and the dipolar and magnetic exchange interactions (*H_u_*) between magnetic compounds [116,117,118]. A large number of partial hysteresis loops (hysterons), called first-order reversal curves, were obtained for the *L*1_0_-MnAlC/α-Fe nanocomposite, as shown in Figure 8a. For this purpose, the sample was exposed to the maximum possible external magnetic field (10 kOe) until the sample reached saturated magnetization and then the field was reduced to the reversal field *H_r_*. Magnetization was measured from this reversal field to the maximum external magnetic field and the FORC was created. A set of FORCs was measured using this method for a series of decreasing reversal fields.

The magnetization in the FORC in the applied *H* field for the reversal field *H_r_* is shown with *M* (*H_r_*, *H*), where *H* ≥ *H_r_* [119,120,121,122]. The FORC distribution can be defined by the following equation.

(3) $$\rho \left(H_{r},H\right)=-\frac{1}{2}\frac{\partial \;2M\;\left(H_{r},H\right)}{\partial \;H_{r}\;\partial \;H}$$

It is known that the FORC distribution becomes non-zero when there is irreversible switching in the magnetization reversal. In addition, the zero FORC distribution is attributed to the occurrence of reversible switching [123,124].

To better illustrate FORC, defining a new set of coordinates can be helpful. For this purpose, the FORC diagram can be rotated from the plane (*H_r_*, *H*) to the plane (*H_c_*, *H_u_*) at an angle of 45°.

(4) $$H_{u}=\frac{H_{r}+H}{2},H_{c}=\frac{H_{r}-H}{2}$$

If each magnetic phase is independently reversed, it is predicted that two peaks corresponding to each of the hard and soft magnetic phases will appear in the FORC diagram. Conversely, when an exchange coupling occurs between the hard and soft magnetic phases, a single peak (two peaks combine at the same peak) must be observed [4,125]. In the FORC diagram, closed lines are usually considered as single-domain particle fingerprints, whereas open lines that diverge toward the *H_u_* axis are fingerprints for multi-domain behavior [126,127].

2D and 3D FORC diagrams of composite NPs as well as its vertical and horizontal profiles and Day plot of single domain are presented in Figure 8. Figure 8b–d shows the FORC contour diagram for F5 nanocomposite. According to Figure 8f, the horizontal profile of F5 sample shows a single peak at *H_c_* (3.5 kOe) which is close to the coercivity of the F5 alloy. The smooth surface of this peak indicates the strong coupling interaction that governs its magnetic behavior. There is a distribution of interaction field *H_u_* (vertical axis) and switching field *H_c_* (horizontal axis), which the former is due to interparticle interactions, and the latter is due to the switching of different particles at different applied field. Peaks in FORC distributions tend to negative interaction fields *H_u_*, a property that is usually associated with the exchange interaction that occurs between magnetic particles [119,124,128]. Figure 8g,h show the interaction field distribution *H_u_* = 0 and *H_u_* = 3.5 kOe which the maximum FORC distribution is concentrated near *H_u_* = −300 Oe and tends to negative *H_u_* field.

The FORC distribution in the Figure 8d is elongated along the central horizontal axis of *H_c_* (horizontally elongated rings), a teardrop-like feature observed in the contour line pattern. Another point is the closed contour lines. These two properties are characteristic of the single-domain particles [123,129,130]. Additionally, the diagram of squareness (*M_rs_*/*M_s_*) versus the remanent coercivity force (*H_cr_*/*H_c_*) is plotted in Figure 8e. This diagram can be used to estimate the type of magnetic domain and shows that the particles size range is in the stable single domain particle region. Single domain particles have a high surface-to-volume ratio, which causes the boundary of the domain regions to disappear and volumetric energies such as de-magnetic energy are increased, hence single-domain particles have high magnetostatic energy [131,132]. This is the reason of the negative value in the δM diagram of Figure 4 for the Mn-Al-C, indicating magnetostatic energy.

Wide distribution on the *H_c_* axis is a statistical representation of the coercivity distribution between domains and has been interpreted as a wide particle size distribution as well as the presence of different anisotropy between particles [130].

Figure 8c shows the FORC distribution of F5 nanocomposite in coordinates (*H_r_*, *H*). For F5 nanocomposite, reversal occurs primarily by rotation of the domain (reverse magnetization nucleation) in the soft phase due to the low *K*. It can be seen from line 1 in Figure 8c. The zero ρ is related to the reversible change in magnetization. After the domain rotation, the beginning of irreversible processes can be observed from the FORC distribution due to decreasing the inverse field. This can be seen by scanning line 2 in Figure 8c. This field corresponds to the nucleation field of the soft phase. However, the soft phase domains begin to rotate toward the external applied field, but the nearest hard phases prevent the soft phase from escaping despite the high uniaxial anisotropic force. The applied inverse field did not have enough energy to overcome the rotation of the magnetization vector within the magnetic domain. The external field has an optional direction (θ angle) relative to the easy direction of the phase particles, which causes some of the magnetism to be reversible and some to be irreversible, i.e., the magnetism has rotated from one stable direction to another stable direction, hence the distribution of the FORC in these areas (between lines 2 and 3) is non-zero.

A more positive distribution of FORC around point 3 indicates the occurrence of an irreversible magnetization process. Between lines 3 and 4, the FORC distribution continues to increase, reaching a maximum (around 3500 Oe), indicating a non-zero FORC distribution. This indicates that the applied inverse field is sufficient to overcome the further rotation of the moments and domains. The domains in the soft phase suddenly begin to exceed the hard phases, leading to the irreversible switching of the hard and soft phases. This is due to the fact that both the soft and hard phases are coupled with each other. Therefore, the magnetism of the particles switches from positive to negative. However, some particles with higher anisotropy still exist. Subsequently, these domains are overcome by increasing the applied inverse field, which leads to a non-zero FORC distribution. Hence, when the nanocomposite reaches its negative saturation, irreversible switching occurs and the FORC distribution is zero.

## 4. Conclusions

The α-Fe NPs with weight percentages of 5, 10, 15, and 20% were added to the hard magnetic phase of Mn-Al-C and the magnetic properties and reverse magnetization behavior of nanostructured bulk magnets were investigated. The demagnetization curve (second quarter of the hysteresis loop) of the sample containing 5 wt. % α-Fe was convex. Whereas, for samples with more iron, the curve was concave. Therefore, Mn-Al-C-0.05 α-Fe sample showed good exchange coupling for hard and soft magnetic phases. In addition, the maximum energy product is (*BH*)*_max_* = 1.6 MGOe, which is an increase of 60% compared to the Mn-Al-C sample. A high value of relative residual (0.61) was obtained for the sample containing 5 wt.% α-Fe, which indicates that the easy magnetization axis direction in many soft phases is aligned with the easy direction in hard phases. Switching field distribution diagrams showed that at high concentrations of soft iron phase, this phase is not associated with good exchange coupling and there are biphase magnetic behaviors. The results of the recoil diagram showed that there is a stronger exchange spring behavior in the Mn-Al-C-0.05 α-Fe sample than in the iron-free sample and the magnetization recovery is increased by adding 5% of the soft phase. FORC analysis also showed that the particles in the Mn-Al-C-0.05 α-Fe sample are single-domain. A major peak for this alloy was observed in the FORC diagram, indicating an exchange coupling that is consistent with the results of Henkel plots and recoil analysis.

## Figures and Tables

**Table 1:** Alloy samples with different α-Fe content.

Sample	Composition
F0	Mn-Al-C
F5	Mn-Al-C-0.05α-Fe
F10	Mn-Al-C-0.1α-Fe
F15	Mn-Al-C-0.15α-Fe
F20	Mn-Al-C-0.2α-Fe

**Table 2:** Properties reported on the Mn-Al alloy, and comparison with the present work.

Composition	M_s_ (emu/g)	M_r_ (emu/g)	H_c_ (kOe)	(BH)_max_ (MGOe)	Method	Ref.
Mn_52_Al_45.7_C_2.3_ + 5 wt.% Fe	76	46	3.3	1.6	VIM + HEBM + mix HEBM + HT	This work
Mn55Al45	75–65	35	3.5		PFC + CR	[21]
Mn53Al45C2		58	~1.9 *		AM and IM + HD	[28]
Mn53.35Al43.65V3	75.8	35	2.13		AM + SRMS + HT	[29]
Mn54Al46	125	~35	2.04	1.42	IM + RMS + HT + BM + SPS	[30]
B-N-doped MnAl	79	40	~3.9 *		IM + HPT	[31]
(Mn0.54Al0.46) 99.8Tb0.2	~31	~14	5.43		AM + MSHT + HEBM	[32]
	79.7	19	12.3		ED + HT	[33]
Mn54Al46	0–55	25–5	3–4.2		GA + SABM	[23]
Mn:Al:C = 54:46:2.44	~91	~39	~2.8–3.4 *		IM + HT + P	[34]
70.01 wt. %Mn, 29.48 wt. %Al and 0.51 wt. %C		93	2.7	7	IM + E+HT	[35]
Mn53.3Al45C1.7/FeCo (95/5 wt. %)	76.43	32.71	~2.77 *	0.7	MS and AM + BM + P and S	[36]
Mn55Al45	118.2	~45	1.5		IM + SRMS	[37]
Mn55Al45C2	108	20	0.7		IM + A	[38]
(Mn0.54Al0.46) 100-xCx, x = 3	86.7	~35	3.26		AM + HEBM	[39]
MnAl/Co-2	52	43	2.752	3.38		[40]
Mn54.2Al43.8C2	111.78	50	1.8	7.8	HEBM + A+aged	[41]
Mn54Al46Cx, x = 2	~125	~42	~2.3 *		IM + CR	[42]
Mn54Al46	~55	~28	~2.5		GA + SABM + A	[43]
Mn54Al46 (MnAlSi0.5)	70.3	33	~4.5 *		IM + SAHEBM	[44]
Mn48Al52	96	~77	~3		MAS + A	[45]
(Mn57Al43) 100C1.19	80	36.70	1.47		GA + mixpolymer + E	[46]
(Mn0·54Al0.46) 98C2	80	~46	1.47		VAM + MS	[47]
Mn54.3Al44C1.7	88	~37	1.6	0.66	AM + cryomilled HEBM+	[25]
Mn1.1Al0.9C0.02	99	29	1.6		Cryomilled + Flash heat	[48]
Mn55Al45	~71	~14	2		AM + MS	[49]
Mn0.55Al0.45C0.02	85	~43	~1.5 *	0.78	MA + A	[50]
Mn54Al46C2.44	~60	~28	~2.9 *		IM + MS + A + P	[51]
MnAlC (70.03 wt.% Mn, 29.28 wt.% Al and 0.69 wt.% C)	95	~40	~2.2 *		SSR + A + HT + HP	[14]
Mn56Al44	63.9	~24	2.8		IM + SC + G	[52]
Mn54Al46	~10–43	~5–25	3–4		GA + SABM + A	[19]
Mn54Al46	22.6	12.9	4.9		GA + MM + A	[18]
MnAl thin film/FeCO (8 nm)	~90	~81	~3.4	4.7	DC MAS + Post A	[53]
Mn53Al45C2	~87	72	~2.85 *	3.0	IM + CW + R	[24]
Mn54Al46	105	~20	~1.2 *		high-frequency VIM + A	[54]
Mn51Al46C3	~44	32	22.83		AM + SABM + A	[55]
MnAlCo	15.1	~5	0.85			[56]
(Mn, Fe) Al	77	~54	3.8	2.0	UHV-MAS + A	[57]
Mn54Al46	50.8	24	~2.8 *		AM + MS + cryomilled HEBM	[58]
Mn57Al43	~62	~35	5.3		AM + MS + A+BM	[15]
(Mn0.55Al0.45) 100C2	83	34	2.8		IM + G + HEBM	[59]
Mn54Al46	~29	16	1.8		AM + HEBM + A	[60]
Mn53Al45C2	98	72	~1.6 *		IM + HE	[61]
Mn54Al46	108	73	~3.7	3.1	AM + SRMS + A	[16]
Mn54Al46	39.8	~23	4.2		GA + ε-phase + MM + ECAE	[17]
Mn54Al46	60	~32	3.62		GA + MM + HT	[62]
	70	~62	~8.1	4.44	MBES + A	[63]
Mn54Al46 + 10 wt%Fe	~16	~6	~3.3 *		IM + mix + HSV BM + A	[14]
	98.3			4.7	M + Q(ε-phase) + A	[64]
Mn68.8Al29.98Ni0.78C0.44	48	~35	1.5		DC MAS	[65]
70.7Mn 28.2Al 1.1 C	85	39	3.4	1.29	MA(HEBM) + CC + HT	[66]
Mn1.074 Al0.871 C0.055	84		2.8	5.5	WE	[67]
71.5 Mn, 27.9Al, 0.6 C	73	0.32	1.32		Alloy + Q + HT	[68]
Mn55Al45	83	49	2.14	1.6	M + rapid Q(ε-phase) + HT	[69]
Mn (56at. %) Al (44at. %)	28	~12	2.44		MA + SPS + Rapid thermal A	[70]
Mn53.3Al45C1.7	~80	54.8	~2.8	~2	AM + HEBM + A	[71]
Mn54Al44C2	84	48	~1.8		IM + MS + M + SPS	[72]
Mn–30 wt.% Al	~60	41.2	3.1	1.59	IM + GA(ε-phase) + BM + HT	[73]
(Mn20Al80) 0.95C0.05		8.8	5.6		MA + PAD + HT	[74]
Mn54Al46	89	45	4.8		AM + HEBM + A	[75]
Mn54Al44C2	92	52	1.7	1.5	VIM + SRMS	[76]
Mn54Al46	87	~38	4.8		AM + HEBM + A	[77]
Mn-29%Al-0.5%C-0.5%Ni		60	1.95	1.43	AM + HEBM + HT	[78]
	~40	24	2.3		MA(HEBM) + HT	[79]
Mn53.5Al44.5C2	82	50	2.2	1.8	AM + HEBM + HT + HD	[80]
Mn54Al44C2	86	41	~1.7 *		M + MS + cryogenic MM + SPS	[81]
	~75	32	2		IM + HD	[82]
Mn54Al46	~75	~41	~3.7 *		AM + VibrationM + post A	[83]
Mn55Al44 + 2.0 wt.% MWCNT	82.3	45.6	3.64	2.26	AM + mix + HEBM + SPS	[84]
Al-55at.%Mn	72.2	~30	~1.5 *		IM + Q (ε-phase) + IFHT	[85]
Mn53.5Al44.5C2	83	51	2.2	1.8	AM + HD	[86]
Mn54Al46	~5	~3	~2.4		MA(HEBM) + SPS	[87]
Mn (56 at. %) and Al (44 at. %)	40.3	~19	2.3		MA + mix + HEBM + SPS	[88]
Mn54Al43C3	~59	~40	3.6		AM + MS(ε phase) + HEBM + HT	[89]
Mn54Al44C2	~90	~46	~1.9 *		IM + MS + A	[90]
Mn0.53 Al 0.46 C0.01	81	28	2.2		MA + M + HT + AA	[91]
50%Mn-50%Al	52	~21	2.6		MA(HEBM) + two-step HT	[92]
	~110	~36	1.95		mix (HEBM) + M+ vibration M + HPS	[93]
Mn54Al44C2	60.34	37.36	3.8		MS + HEBM + A	[94]
Mn0.53Al0.46C0.01	72	37	1.9	0.6	MA(HEBM) + A	[95]
Mn53.3Al45C1.7	127	46	1.54	1.53	AM + SRMS	[96]
70.5 wt.%Mn-29.5 wt.%A1, 0.8 wt.% C		43	1.5	1.2	IM + Q (ε-phase) + BM + A + CC + S	[97]
		57	1.83	2.1	IM + SRMS + HT	[98]

* Unit conversion from T to MGOe according to 1T = 0.01 MGOe. Abbreviations: M = melt; AM = arc melt; VAM = vacuum arc melting; IM = induction melt; VIM = vacuum induction melt; HD = hot-deformation; SRMS = single-roller melt-spinning; RMS = roller melt-spinning; MS = melt-spinning; HT = heat treatment; HEBM = high energy ball milling; SABM = surfactant-assisted ball milled; BM = ball milling; SPS = spark plasma sintering; HPT = high pressure torsion; HPS = high-pressure synthesis; HP= high press; P = press; ED = electrodeposite; GA = Gas-atomized; HE= hot extruded; WE = warm extruded; E = extruded; A = aged; MA = mechanical alloying; MM= Mechanical milling; S = sinter; A = annealing; CR = cold rolling; CC = cold compact; CW = cold worked; SAHEBM = salt-assisted high-energy ball milling; MAS = magnetron sputtering; SSR = solid-state reaction; SC = strip casting; G = grind; R = recovered; ECAE = equal channel angular extrusion; MBES = molecular-beam epitaxy system; HSV BM = high-speed vibration ball mill; Q = quench; PAD = plasma arc discharge; IFHT = In-field heat treatment; AA = ambient aging; PFC = planar flow casting.

## Data Availability

The data presented in this study are available on request from the corresponding author or first author.

## Supplementary Materials

The following supporting information can be downloaded at: https://www.mdpi.com/article/10.3390/nano12193303/s1, Figure S1: SEM images (in different magnification) and size distribution of Mn_52_Al_45.7_C_2.3_ nano powders.
