# Supplementary material for: Reverse Magnetization Behavior Investigation of Mn-Al-C-(α-Fe) Nanocomposite Alloys with Different α-Fe Content Using First-Order Reversal Curves Analysis
Source: Nanomaterials (Basel). 2022 Sep 22;12(19):3303. doi: 10.3390/nano12193303 (PMC9565730; doi:10.3390/nano12193303)
Supplement: Supplementary file 1 [file nanomaterials-12-03303-s001.zip › nanomaterials-1900577-supplementary.pdf]

Supplementary Materials

Reverse Magnetization Behavior Investigation of Mn-Al-C-(α-Fe) Nanocomposite Alloys with Different α-Fe Content Using First-Order Reversal Curves Analysis

$Mn_{52}Al_{45.7}C_{2.3}$ particle size measurement

The average particle size was obtained using ImageJ tool on SEM images. The measurments were performed on more than 400 particles and the average particle size was about 100 nm. The SEM images and size distribution of $Mn_{52}Al_{45.7}C_{2.3}$ nano powders are shown in Figure S1.

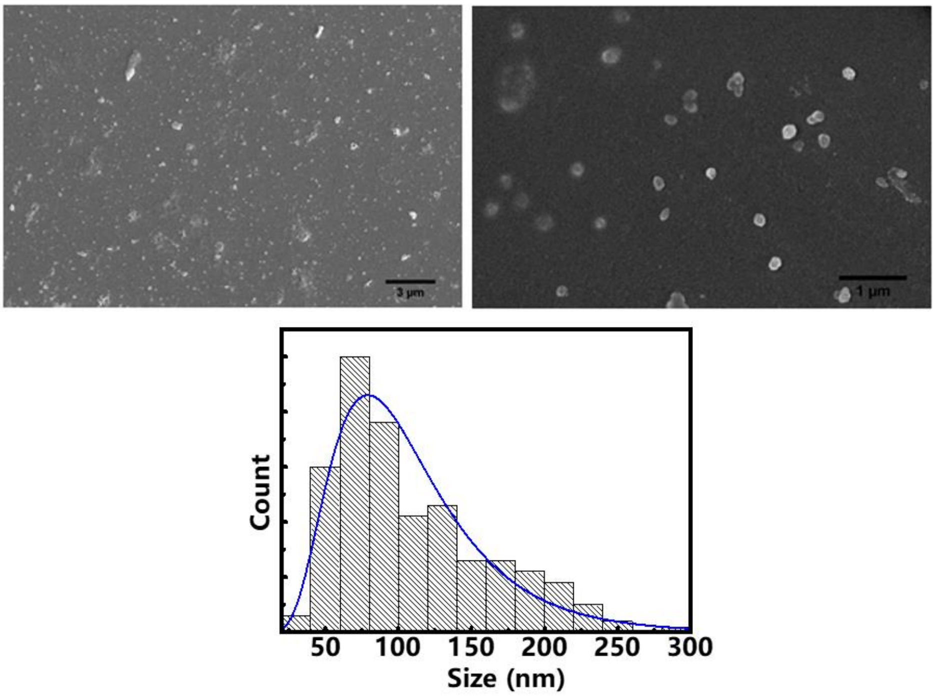

Figure S1. SEM images (in different magnification) and size distribution of $Mn_{52}Al_{45.7}C_{2.3}$ nano powders.
